# MicroRNA Landscape in Endometrial Carcinomas in an Asian population: Unraveling Subtype-Specific Signatures

**DOI:** 10.3390/cancers15215260

**Published:** 2023-11-02

**Authors:** Gideon Ze Lin Tan, Sai Mun Leong, Yu Jin, Chik Hong Kuick, Jeremy Joon Keat Chee, San Zeng Low, Ling-Wen Ding, He Cheng, Diana Lim, Susan Swee-Shan Hue

**Affiliations:** 1 Department of Pathology, National University Hospital, Singapore 118177, Singapore; gideon_tan@nuhs.edu.sg (G.Z.L.T.); sanzeng.low@mountelizabeth.com.sg (S.Z.L.); diana_gz_lim@nuhs.edu.sg (D.L.); 2 Department of Pathology, Yong Loo Lin School of Medicine, National University of Singapore, Singapore 119077, Singapore; patlsm@nus.edu.sg (S.M.L.);; 3 MiRXES Pte Ltd., Singapore 618305, Singaporehecheng@mirxes.com (H.C.); 4 Department of Pathology and Laboratory Medicine, KK Women’s and Children’s Hospital, Singapore 229899, Singapore; 5 Cancer Science Institute of Singapore, National University of Singapore, Singapore 117599, Singapore

**Keywords:** endometrial carcinoma, micro-RNA, pathology

## Abstract

**Simple Summary:**

In this paper, we analyzed the expression of microRNAs in endometrial carcinomas, measuring their expression in histological subtypes, molecular subtypes, and tumors with *CTNNB1* mutations. Our findings provide an insight into different microRNA expression profiles in the different subtypes of endometrial carcinoma in an Asian population and may have implications for the diagnosis, treatment, and prognosis of endometrial carcinoma.

**Abstract:**

MicroRNAs (MiRNAs) are small, non-coding RNA molecules that function in RNA silencing and post-transcriptional regulation of gene expression. We analyzed the differential expression of miRNAs in 119 endometrial carcinomas, measuring their expression in histological subtypes, molecular subtypes, and tumors with *CTNNB1* mutations. Tumors were subdivided into histological and molecular subtypes as defined by The Cancer Genome Atlas. The expression levels of 352 miRNAs were quantified using the PanoramiR panel. Mir-449a, mir-449b-5p, and mir-449c-5p were the top three miRNAs showing increased expression in both endometrioid and de-differentiated carcinomas but were not significantly increased in serous and clear cell carcinomas. The miRNAs with the most increased expression in serous and clear cell carcinomas were miR-9-3p and miR-375, respectively. We also identified 62 differentially expressed miRNAs among different molecular subtypes. Using sequential forward selection, we built subtype classification models for some molecular subtypes of endometrial carcinoma, comprising 5 miRNAs for MMR-deficient tumors, 10 miRNAs for p53-mutated tumors, and 3 miRNAs for *CTNNB1*-mutated tumors, with areas under curves of 0.75, 0.85, and 0.78, respectively. Our findings confirm the differential expression of miRNAs between various endometrial carcinoma subtypes and may have implications for the development of diagnostic and prognostic tools.

## 1. Introduction

Endometrial carcinoma (EC) is the second most common gynecologic cancer worldwide [1]. Current EC classification relies heavily on morphological features, which can be subjective, lack reproducibility, and may be inadequate in capturing the biological diversity of the tumors. The Cancer Genome Atlas (TCGA) has classified endometrial carcinoma into four molecular subtypes that transcend histological features—(1) *POLE* ultramutated, (2) microsatellite instability hypermutated, (3) copy number low, and (4) copy number high [2]. This classification has shown a significant correlation with patient outcomes, helping to guide treatment.

MicroRNAs (miRNAs) are short, evolutionarily conserved, non-coding RNA averaging 18–25 nucleotides in length [3,4]. They account for more than 3% of all human genes and play an important role in post-transcriptional regulation of gene expression through messenger RNA (mRNA) degradation or translational repression [5,6,7]. Apart from their involvement in normal physiological processes, miRNAs have also been implicated in many oncogenic processes including cellular differentiation, proliferation, apoptosis, metastasis, and angiogenesis [8,9,10,11]. MiRNAs are known to demonstrate tissue and cell type-specific expression [12,13]. Despite some dysregulation in diseases and neoplastic transformation, it appears that tissue-specific expression patterns are still preserved in the corresponding tumor tissues, resulting in tumor-specific miRNA signatures [14,15,16,17,18,19]. In view of their role in oncogenesis and their distinct expression profiles in tumors, miRNA expression profiles are attractive candidates to aid tumor classification in line with their role in cellular lineage, differentiation state, and molecular alterations. Another appealing attribute of miRNAs as biomarkers for clinical use is their high stability in tissues and biofluids [20]. MiRNAs are well-preserved and can be robustly detected in routinely processed formalin-fixed paraffin-embedded (FFPE) tissues which have a long storage time, rendering them particularly useful for applications in routine clinical practice [21].

Several studies have already demonstrated the dysregulation of miRNAs in endometrial carcinoma. In our study, we further explore the differential expression of miRNAs between histological subtypes and between molecular subtypes of endometrial carcinoma. To do so, we performed miRNA expression profiling on FFPE tissue samples of endometrioid carcinoma, serous carcinoma, clear cell carcinoma, and de-differentiated carcinoma. We sought to identify distinctive miRNA signatures that could be used as a diagnostic adjunct in the classification of these tumors and to help better understand the biology of these tumors.

Catenin Beta 1 (*CTNNB1*) mutations are typically found in low-grade carcinomas with endometrioid histology [22]. While these tumors tend to be early stage with lower rates of myometrial and lymphovascular invasion, *CTNNB1*-mutated cancers show increased rates of recurrence [22,23]. In our paper, we also analyzed the miRNA expression of tumors with *CTNNB1* mutations in an attempt to elucidate important biological pathways.

## 2. Materials and Methods

### 2.1. Patients and Cases

Following approval by the institution’s Institutional Review Board, FFPE tissue samples of endometrial carcinoma from 119 patients over the period 2008–2018 were obtained from the Department of Pathology, National University Hospital (NUH), Singapore. The inclusion and exclusion criteria for the cases are shown in Table 1. All cases had undergone total hysterectomy, and a further 77 and 23 patients had also undergone pelvic and para-aortic lymphadenectomy, respectively.

### 2.2. Classification According to Histological Subtypes

**Figure 1:**
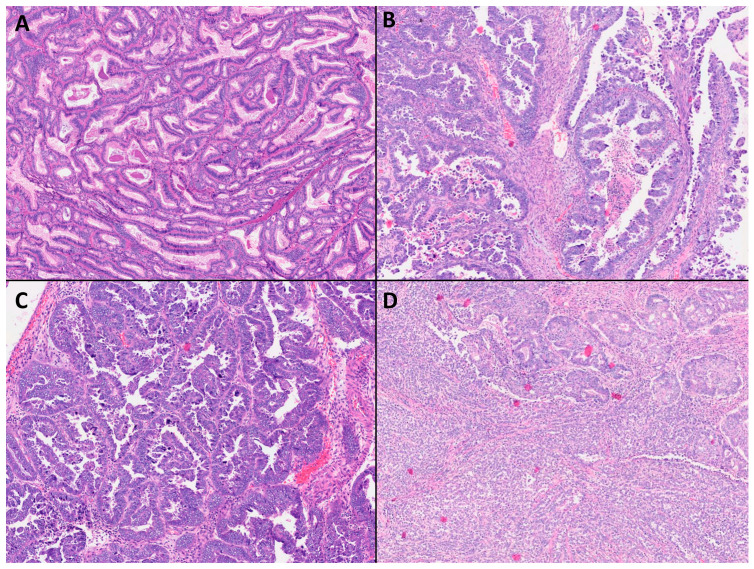
Histological images (Original magnifications, ×60) from cases of (**A**) endometrioid carcinoma; (**B**) clear cell carcinoma; (**C**) serous carcinoma; and (**D**) de-differentiated carcinoma.

The breakdown of the histological subtypes of the cases is shown in Table 2, and representative histological images of the different subtypes are shown in Figure 1. All cases had been previously reported in our institution, and all cases were also reviewed again by the department's gynecologic pathologist to verify the diagnosis.

### 2.3. Classification According to Molecular Subtypes

**Figure 2:**
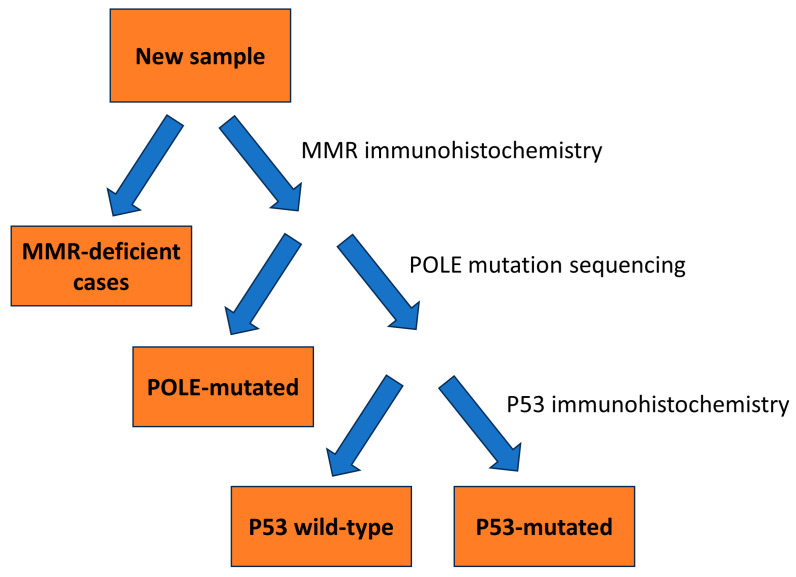
Flowchart for classification of tumors according to molecular subtype.

Of the 119 specimens, 102 were further classified into their molecular subtypes, according to The Cancer Genome Atlas (TCGA). The characteristics of the 102 cases are shown in Table 3 and Table 4. In order to categorize tumors into their respective molecular subtypes, we utilized a classification model proposed by A. Talhouk et al. (Figure 2) [24]. In this model, mismatch repair (MMR) immunohistochemistry was used as the surrogate for MSI mutation status, and p53 immunohistochemistry was used as a surrogate to determine copy-number status, with p53 mutant tumors representing the copy-number-high subgroup, and p53 wild type representing the group with no specific molecular profile, or copy-number-low group. We utilized this model due to its cost-effectiveness and accuracy—the authors had demonstrated that it replicated the survival curves of the various molecular subtypes as laid out by TCGA. MMR immunohistochemistry was performed on all 119 cases, while 92 cases underwent genetic sequencing.

### 2.4. DNA Extraction

DNA was extracted from all the FFPE tissues using the QIAamp^®^ DNA FFPE Tissue kit (Qiagen, Germantown, MD, USA). The tissues were cut 10 microns thick. The cut tissues were placed in Eppendorf tubes and kept at 4 °C until the time of extraction. The DNA extraction was performed according to the manufacturer’s guidelines. After extraction, the concentration of the DNA (A260/280) was measured using the Infinite M200 spectrophotometer (Tecan, Morrisville, NC, USA).

### 2.5. Library Preparation for Sequencing

The library preparation for the samples was performed using a SureSelectXT Low Input Target Enrichment System for Illumina Paired-End Multiplexed Sequencing Library according to the manufacturer’s instructions. DNA was sheared using a Covaris sonicator. Five µL of DNA from each sample was used for gel electrophoresis QC. At least 100 ng of DNA or more from each sample was used for library preparation. The libraries were then quantified and sequenced by Novogene, using HiSeqPE150.

### 2.6. DNA Sequencing

This was performed by an external vendor using Agilent SureSelect XT Low input sequencing. Ninety-two cases underwent DNA sequencing.

### 2.7. CTNNB1 Mutations

The 92 tumors sent for DNA sequencing were also sequenced for *CTNNB1* mutations. The histological subtypes of the cases with *CTNNB1* mutations are shown in Table 5.

### 2.8. POLE Mutation Analysis

Sequencing was undertaken on 92 specimens to detect mutations in the *POLE* exonuclease domain to identify *POLE* ultra-mutated/*POLE*-mutated tumors.

### 2.9. Constructing the Tissue Microarray (TMA)

Morphologically representative regions of each tumor were marked out by a pathologist. For each tumor, two core biopsies of 2 mm diameter each were taken from within the tumor area. Four μm thick sections were cut from the TMAs.

### 2.10. Immunohistochemistry for DNA Mismatch Repair and p53 Staining

The TMAs were stained with antibodies against the MMR protein products: MLH1 (clone M1, Ventana, Export, PA, USA, predilute), MSH2 (clone G219-1129, Ventana, Export, PA, USA, predilute), MSH6 (clone SP93, Ventana, Oro Valley, AZ, USA, predilute), and PMS2 (clone Al6-4, Ventana, Oro Valley, AZ, USA, predilute), and against p53 protein (clone DO-7, DAKO, Santa Clara, CA, USA, 1:100).

### 2.11. Analysis of TMA

**Figure 3:**
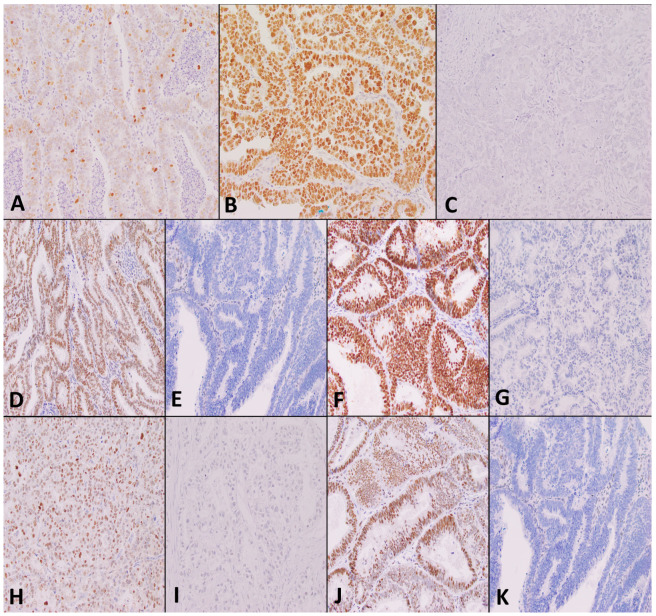
Analysis of tumor samples stained by immunohistochemistry (Original magnifications, ×200). Figures depict (**A**) p53 wild type; (**B**) p53 mutant over-expressed; (**C**) p53 mutant null pattern of expression; (**D**) MSH2 intact; (**E**) MSH2 loss; (**F**) MSH6 intact (**G**) MSH6 loss (**H**) PMS2 intact (**I**) PMS2 loss (**J**) MLH1 intact (**K**) MLH1 loss.

Two independent observers (G.T. and S.H.) scored the immunohistochemistry-stained slides without prior knowledge of clinicopathological information. Images of the TMAs are shown in Figure 3.

MMR protein status was considered deficient (MMRd) when the tumor showed a complete loss of nuclear expression of any of the MMR proteins (MLH1, PMS2, MSH2, MSH6). Positive controls were checked to ensure they showed retained expression. 

P53 was assessed as wild type when the tumor showed a heterogenous pattern of staining, and mutant type when the tumor showed more than 80% strong diffuse nuclear staining, complete absence of nuclear staining, or moderate to strong cytoplasmic positivity, as outlined by guidelines recommended at the 2020 USCAP annual meaning.

### 2.12. RNA Extraction

To investigate the differential expression of MiRNAs between tumor types, we performed an analysis of miRNA levels using the ID3EAL™ PanoramiR miRNA Knowledge Panel (MiRXES, Singapore). A total of 352 miRNAs were analyzed.

To select the miRNas to be tested, miRNAs were sorted according to their frequency of appearance in Pubmed, and cross-referenced to miRbase version 21. The overlapping ones were chosen. MiRNAs with high Guanine and cytosine (GC) content and repeated sequences were excluded. The miRNAs to be tested were fitted into a 384-well plate format, which also included 32 synthetic controls for reverse transcription polymerase chain reaction (RT-qPCR) and isolations. A total of 352 miRNAs was tested—the list of miRNAs tested is shown in Table S1. 

### 2.13. Statistical Analysis

In order to elucidate differential expressions of miRNA between tumor subtypes, the level of expression of each miRNA was analyzed for each variable, taking the other samples as the control group. Student’s *t*-test was performed. The difference in miRNA expression was calculated, the labels were shuffled, and the *t*-test was repeated 1000 times to calculate the permutated *p*-value. MiRNAs with a significant difference in expression, with a permutated *p*-value of less than 0.05, were identified. This was carried out for both histological and molecular subtypes of endometrial carcinoma.

For molecular subtypes of endometrial carcinoma, we further built a multi-miRNA model using sequential forward selection. Using n-fold cross-validation, we identified the most informative miRNAs, adding them to the model. We repeated this 50 times to obtain the average area under the curve (AUC).

## 3. Results

### 3.1. Differential Expression of miRNAs between Histological Subtypes

**Figure 4:**
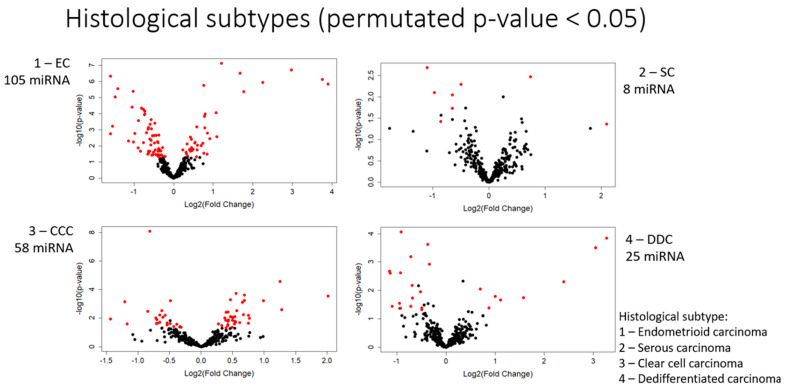
Number of miRNAs differentially expressed in each histological subtype.

We first examined the expression of miRNAs in the histological subtypes of endometrial carcinoma. Significantly differentially expressed miRNAs, with a permutated *p*-value of less than 0.05, were identified. Those with the greatest differential expressions between subtypes, with a fold change of greater than 0.5, are shown in Table 6. The number of differentially expressed miRNAs for each subtype is shown in Figure 4. 

#### 3.1.1. Endometrioid Carcinoma

Endometrioid carcinomas had the greatest number of differentially expressed miRNAs among all histological subtypes studied. A total of 105 miRNAs showed differential expression in endometrioid carcinoma, with 40 showing an increase in expression and 65 showing a decrease. Of these, 28 showed a fold change of greater than 0.5, the majority of which showed increased expression. Among the most differentially expressed miRNAs were MiR-449a, miR-449b-5p, and miR-449c-5p, with fold changes of 14.94, 13.50, and 7.87, respectively.

#### 3.1.2. Serous Carcinoma

In serous carcinomas, eight miRNAs showed differential expression, with two showing an increase in expression and six showing a decrease. Of these, three showed a fold change of greater than 0.5. MiR-9-3p showed the most significant differential expression, with a fold change of 4.26.

#### 3.1.3. Clear Cell Carcinoma

A total of 58 miRNAs showed differential expression in clear cell carcinoma, with 35 showing an increase in expression and 23 showing a decrease. Of these miRNAs, 13 showed a fold change of greater than 0.5. Mir-375, Mir-638, and Mir-1915-3p showed the greatest increases, with fold changes of 4.05, 2.39, and 2.43, respectively.

#### 3.1.4. De-Differentiated Carcinoma

A total of 25 miRNAs showed differential expression in de-differentiated carcinoma, with 8 showing an increase in expression and 17 showing a decrease. Of these miRNAs, 11 showed a fold change of greater than 0.5. Mir 449a, mir 449b-5p, and mir-449c-5p showed the greatest increases, with fold changes of 9.68, 8.30, and 5.28, respectively.

### 3.2. Differential Expression of MiRNAs between Molecular Subtypes

**Figure 5:**
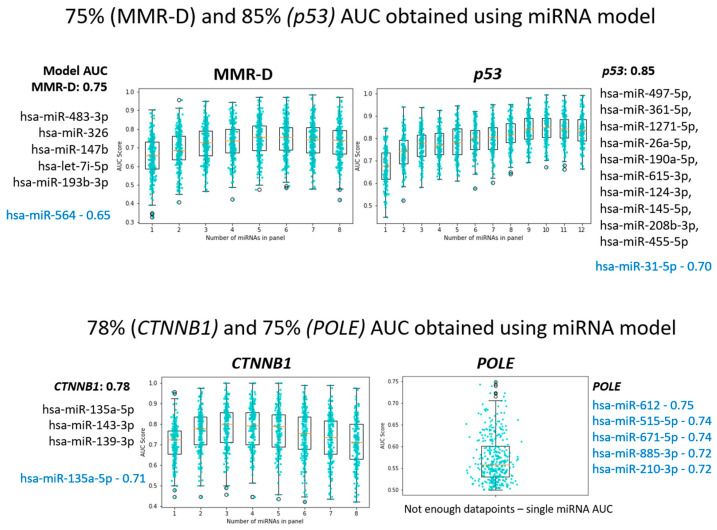
Subtype classification models for the molecular subtypes of endometrial carcinoma. Sequential forward selection was used to build subtype classification models. Model AUC is superior to single feature AUC for classification.

Differing miRNA expression levels were also detected when tumors were classified according to molecular subtypes. Using sequential forward selection, we built subtype classification models for the molecular subtypes of endometrial carcinoma, comprising 5 miRNAs for MMR-deficient tumors and 10 miRNAs for p53 mutated tumors, with AUCs of 0.75 and 0.85, respectively (Figure 5).

It is noted, though, that the differential expression of miRNAs between molecular subtypes is less significant than the differential expression between histological subtypes. Fewer miRNAs were differentially expressed, and the fold changes between subtypes were also lower. The most differentially expressed miRNAs for each molecular subtype are shown in Table 7.

#### 3.2.1. MMR-Deficient Group

We built a 5-miRNA subtype classification model for MMR-deficient endometrial carcinomas. The five miRNAs miR-483-3p, miR-326, miR-147b, let-7i-5p, and miR-193b-3p had an AUC of 0.75 (Figure 5).

When analyzed for differential expression of individual miRNAs, the MMR-deficient group showed the least differential expression of miRNAs among molecular subtypes. While there were 19 miRNAs with differential expression, the fold changes were only slight, ranging from 0.1 to 0.4. No miRNA showed a fold change of greater than 0.5. Of note, miR-483-3p had a fold change of 1.34 in the MMR-deficient group but was decreased in the *POLE*-ultra-mutated group.

#### 3.2.2. POLE-Ultra-Mutated Group

When analyzed for differential expression of individual miRNAs, 21 miRNAs showed differential expression in the *POLE*-ultra-mutated group, with 3 showing an increase in expression and 18 showing a decrease. MiR-139-5p showed the most significant increase, with a fold change of 1.59, while miR-515-5p and miR-210-3p showed the greatest decrease in expression with fold changes of 0.31 and 0.49, respectively.

#### 3.2.3. P53 Mutant Group (Surrogate for Copy Number High)

We built a subtype classification model for p53 mutated endometrial carcinomas. The ten miRNAs (hsa-miR-497-5p, hsa-miR-361-5p, hsa-miR-1271-5p, hsa-miR-26a-5p, hsa-miR-190a-5p, hsa-miR-615-3p, hsa-miR-124-3p, hsa-miR-145-5p, hsa-miR-208b-3p, hsa-miR-455-5p) had an AUC of 0.85.

When analyzed for differential expression of individual miRNAs, 20 miRNAs showed differential expression in the p53 mutant group, with 7 showing an increase in expression and 13 showing a decrease. Only 3 of these 20 miRNAs showed fold changes of greater than 0.5. MiR-34c-5p and miR-133b showed the most significant increases in expression, with fold changes of 2.04 and 1.53, respectively, while miR-31-5p showed the greatest decrease in expression, with a fold change of 0.30.

#### 3.2.4. P53 Wild Type (Surrogate for Copy Number Low)

A total of 13 miRNAs showed differential expression in the p53 wild-type group, with 6 showing an increase in expression and 7 showing a decrease. MiR-205-5p and miR-200a-3p showed the most significant increases in expression, with fold changes of 2.07 and 1.51, respectively, while miR-31-5p showed the greatest decrease in expression, with a fold change of 0.34. No other miRNAs showed a fold change of greater than 0.5.

#### 3.2.5. *CTNNB1* Mutation

Greater proportions of tumors in our study with *CTNNB1* mutations showed endometrioid morphology and had a lower grade (Table 5), concordant with the existing literature [22,23].

We built a 3-miRNA, multi-miRNA subtype classification model for *CTNNB1* mutated tumors, with an AUC of 0.78 (Figure 5). These miRNAs comprised miR-135a-5p, miR-143-3p, and miR-139-3p.

When analyzed for differential expression of individual miRNAs, seven miRNAs showed differential expression in tumors that harbored *CTNNB1* mutations (Table 8). Of these, miR-203a-3p showed the greatest difference in expression, with a fold change of 1.71.

#### 3.2.6. MirPath Analysis

**Figure 6:**
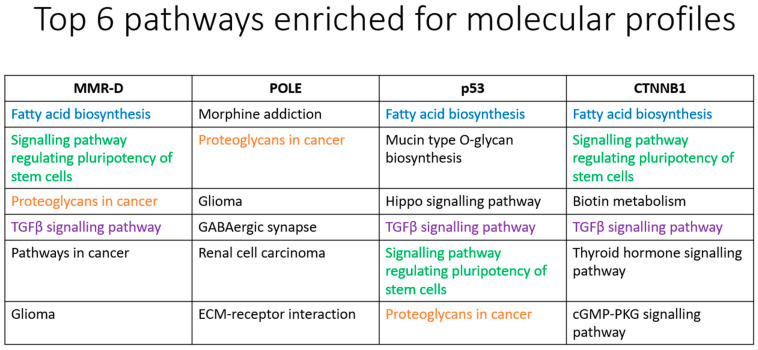
Top 6 pathways enriched for molecular profiles of endometrial carcinoma.

Using miRNAs that had significant (permutated *p*-value < 0.05) differential expression levels, we performed KEGG pathway analysis on mirPath (https://dianalab.e-ce.uth.gr/html/mirpathv3/index.php?r=mirpath, accessed on 12 May 2022) and have presented the top six molecular pathways enriched for each molecular subtype (Figure 6). Among the four molecular subtypes examined, *POLE* subtype appears to be markedly different from the other three in terms of the KEGG-analyzed signaling pathways. The result may provide insights into the distinct molecular characteristics of endometrial carcinoma subtypes and their associated signaling pathways, which may have implications for targeted therapeutic approaches.

## 4. Discussion

Recent studies have shed light on the expression of miRNAs in endometrial carcinomas. Firstly, various studies have confirmed the differential expression of miRNAs in endometrial carcinomas when compared to normal endometrium [25,26,27,28,29,30]. Studies have also demonstrated upregulation of the miRNA-200 family (miR-200a, -200b, -200c, -141, -429) in cases of endometrioid carcinoma [25,26]. Secondly, miRNA expression has been shown to correlate with the International Federation of Obstetrics and Gynecology (FIGO) stage, nodal metastasis, disease recurrence rates, and myometrial invasion, suggesting that miRNAs may be discriminatory in early versus advanced disease and show promising utility in evaluating a patient’s prognosis [26,27,28]. However, few studies have ventured into the study of differential miRNA expression in tumor types. Only three other studies have studied the differential expression of miRNAs in histological subtypes of endometrial carcinoma, and only one has compared the differential expression of miRNAs in MMR-deficient vs. MMR-intact cancers [28,29,30]. No studies to date have evaluated the differential expression of miRNA in other molecular subtypes of endometrial carcinoma such as those with *POLE* and p53 mutation. In light of this knowledge gap, we have developed miRNA classification models for MMR-deficient and p53 mutated/copy-number-high tumors and have evaluated the differential expression of miRNA in molecular subtypes and some histological subtypes. The results from our study may have several clinical benefits. Histological subtypes of endometrial carcinomas can sometimes be difficult to distinguish on histology alone. By demonstrating significant differentially expressed miRNAs between histological subtypes, we move one step closer to developing another diagnostic adjunct for endometrial carcinoma. As they have been shown to correlate with prognostic variables, and in our study, molecular subtype, miRNAs show great promise as prognostic markers as well. Classifying endometrial carcinomas according to molecular subtype may be costly as DNA sequencing is required. MiRNAs may be able to provide an alternative mode of classifying endometrial tumors.

### 4.1. Limitations of the Paper

One limitation of our study is the use of immunohistochemistry TMAs in the classification of molecular subtypes. The immunohistochemical staining in the small tissue sample may not be representative of the entire tumor, as some tumors may demonstrate heterogenous staining qualities. In our study, the proportion of MMR-deficient tumors is greater than expected. This is partly due to the fact that all tumors were tested for MMR, while only 92 of the tumors underwent molecular sequencing. It is also possible that the use of TMAs rather than full-face section slides led to an over-estimation of MMR-deficient tumors.

In calculating differences in miRNA expression, we utilized fold change. A disadvantage of using fold change is that, being an expression of a ratio, it is not able to identify the actual quantitative differences in mRNA expression—large quantitative differences in mRNA expression may be missed at high expression levels. 

### 4.2. Evaluation of miRNA Expression with Molecular Subtype

The Cancer Genome Atlas (TCGA) has identified four groups of carcinomas: Group 1, with *POLE* mutations, is associated with a good prognosis; Group 2, with microsatellite instability, is associated with an intermediate prognosis; Group 3, showing low copy-number alterations, is also associated with an intermediate prognosis; and Group 4, with high copy-number alterations and *TP53* mutations, is associated with a poor prognosis [2]. This classification by TCGA is important for the treatment and prognostication of patients and overcomes the problem of inter-observer variability in typing endometrial carcinomas, especially high-grade endometrial carcinomas [2]. Determination of molecular subtypes is very costly, however, requiring advanced methods including genomic sequencing, MSI assays, and single nucleotide polymorphism arrays. This is not feasible in most laboratories. Talhouk et al. developed a model for molecular classification utilizing immunohistochemistry for MMR, p53, and *POLE* mutational analysis, demonstrating that the subtypes produced by this model replicated the survival curves of TCGA subtypes [24]. We have used this model for the molecular classification of tumors in our study. While cost-effective and accurate, this model in its current state has some limitations. Firstly, the goal of the study was to replicate survival curves of molecular subtypes as outlined by TCGA, rather than definitively test for the genetic mutations—given the relatively small sample size, the authors themselves acknowledge that further validation of their model is warranted. Also, the optimal order of testing, as outlined in Figure 2, remains to be determined. Lastly, while the model significantly reduces the cost of genetic testing, *POLE* mutational analysis is still required—not all laboratories may have the testing capabilities.

By evaluating the differential miRNA expression between molecular subgroups, our study provides insight into the differential miRNA expression between molecular subtypes, and at the same time, complements the study by Talhouk et al.

### 4.3. MMR-Deficient Tumors

We built a multi-miRNA subtype classification model for MMR-deficient tumors, comprising the miRNAs miR-483-3p, miR-326, miR-147b, let-7i-5p, and miR-193b-3p—this model had an area under the curve of 0.75. With regards to individual miRNAs, we found 19 differentially expressed miRNAs between MMR-intact and MMR-deficient tumors. While it is worth noting that the difference was not marked—no miRNAs were differentially expressed at greater than 50%, the differences were nonetheless significant for a permutated *p*-value of <0.05. We are the first to report the association of several of these miRNAs with MMR-deficient endometrial carcinoma (miR-483-3p, miR-885-5p, miR-1915-3p, miR-564, miR-196a-5p, and miR-16-5p showed increased expression, and miR-142-5p and miR-301a-3p showed decreased expression). Some of these miRNAs have previously been reported to be associated with other carcinoma subtypes. MiR-885-5p has been previously reported to be increased in serous carcinomas of the endometrium and is also associated with clear cell renal cell carcinoma and gastric carcinoma [30,31,32]. MiR-1915-3p and miR-564 have been reported to be associated with gastric carcinoma, breast carcinoma, and non-small cell lung carcinoma [33,34,35].

Only one other study thus far has evaluated miRNA expression in MMR-intact vs. MMR-deficient endometrial tumors. Cohn et al. compared the expression of miRNAs between MMR-intact and MMR-deficient endometrial carcinomas of endometrioid subtype, and found six miRNAs (mir-29a, -126, -1-2, -143, -125b, and -133) that had at least a twofold reduction in expression [28]. Three of these under-expressed miRNAs targeted the DNA MMR genes MLH1 or MSH2. Furthermore, two of the miRNAs were known to target DNMT3A, the gene regulating epigenetic modulation and underlying DNA MMR in sporadic endometrial carcinoma. Notably, these miRNAs are different from those found in our study. We found, instead, six other miRNAs with increased expression (miR-483-3p, -885-5p, -1915-3p, -564, -196a-5p, -16-5p), and two with decreased expression (miR-142-5p, -301a-3p). None of these miRNAs showed greater than 50% differential expression. Such a difference between Cohn’s results and ours can possibly be explained by the difference in tumor types among the tumors analyzed. While the study group in Cohn’s study comprised 108 endometrioid and 13 serous carcinomas, our study group comprised a greater variety of tumors including endometrioid, serous, clear cell, and de-differentiated carcinomas. Another possible reason could be the difference in demographics—while most of our patients are Asian, the patients in Cohn’s study were from an American hospital.

### 4.4. POLE-Ultra-Mutated Tumors

In *POLE*-ultra-mutated tumors, miRNAs that showed the greatest increase in expression were miR-139-5p, miR342-3p, and miR-374-5p. Notably, all three miRNAs have been associated with tumor-suppressing effects. Mir-139-5p has been shown to be downregulated in endometrial carcinoma compared to non-tumor tissues [36]. It targets the *HOXA10* transcript and suppresses endometrial carcinoma cell growth and migration [36]. Overexpression of miR-342-3p has been shown to suppress proliferation, migration, and invasion by targeting *FOXM1* in cervical cancer and has also been shown to suppress hepatocellular carcinoma proliferation through the inhibition of the IGF-1R-mediated Warburg effect [37,38]. Mir-374-5p has been reported to inhibit non-small cell lung cancer proliferation and migration via targeting of *NCK1* [39]. These findings are significant and interesting because *POLE*-ultra-mutated tumors have the best prognosis among the four molecular subtypes—increased expression of miRNAs which contribute toward tumor suppression may thus be a noteworthy feature of *POLE*-ultra-mutated tumors based on our study.

### 4.5. P53 Mutation (Surrogate for Copy Number High)

In tumors with p53 mutation, miR-205-5p and miR-200a-3p showed the most significant increases in expression, with fold changes of 2.07 and 1.51, respectively.

There have been three previous studies on miR-205 in endometrial carcinomas. One study found miR-205 to show increased expression in endometrial tumors as compared to normal endometrium, and high levels of miR-205 were correlated with poor patient survival [40]. Mir-205 has also been shown to promote tumor proliferation by targeting estrogen-related receptor-γ in endometrial carcinoma and contributes to paclitaxel resistance and progression of endometrial carcinoma by downregulating *FOXO1*, a tumor suppressor [41,42].

An increase in the expression of miR-200a-3p has likewise been shown to promote cancer growth. In ovarian cancers, miR-200a-3p is shown to possess oncogenic potential, possibly by modulating Protocadherin 9, and increased expressions were associated with increased tumor size and metastasis [43]. MiR-200-a-3p has also been shown to facilitate bladder cancer cell proliferation, metastasis of non-small cell lung cancer cells, and proliferation of esophageal cancer cells by targeting the *A20* gene, downregulating *SOX17*, and by post-transcriptionally regulating cytoplasmic collapsing response mediator protein-1, respectively [44,45,46].

These findings are significant because p53 copy-number-high tumors have a poor prognosis compared to *POLE*-ultra-mutated tumors. In our study, we have elucidated a possible biological explanation—while *POLE*-ultra-mutated tumors are associated with increased levels of miRNAs that serve to suppress tumor growth, p53 mutated tumors are associated with increased levels of miRNAs that promote tumor growth and metastasis.

### 4.6. Evaluation of miRNA Expression with CTNNB1 Mutation

We also built a multi-miRNA expression model for *CTNNB1* mutated cancers, with an AUC of 0.78.

MiR-203a-3p showed the greatest difference in expression, with a fold change of 1.71. Two studies have demonstrated increased miR-203a expression in colorectal carcinomas [47,48]. The study by Lin Chen et al. further showed that the overexpression of MiR-203a-3p suppressed Phosphodiesterase 4D (PDE4D), resulting in increased expression levels of beta-catenin, c-Myc, and cyclin D1, in turn promoting colorectal carcinoma cell proliferation and migration [48].

MiR-499a-5p showed the greatest decrease in expression, with a fold change of 0.47. miR-499a-5p has been found to have tumor-suppressor functions and is downregulated in endometrial carcinoma tissue as compared to adjacent normal tissue [49]. Down-regulation of miR-499a-5p has also been shown to predict a poor prognosis in non-small cell lung carcinoma [50].

MiR-184 was the next most downregulated miRNA in cancers with *CTNNB1* mutations, with a fold change of 0.56. This finding is in concordance with other studies, such as the study by Zhen Chen et al., which found miR-184 to be significantly downregulated in endometrial carcinoma tissues as compared to normal tissues [51]. MiR-184 has also been identified as a tumor suppressor in renal cell carcinoma [52].

In our study, we demonstrate that the differential expression of miRNAs exists even between endometrial carcinomas with and without *CTNNB1* mutations.

### 4.7. MirPath Analysis

In this study, we identified intriguing patterns in subtypes of endometrial carcinoma and their associated signaling pathways. Cancers with three specific mutation profiles (MMD-R, p53, *CTNNB1*) displayed commonality in three pathways, including fatty acid biosynthesis, the pluripotency of stem cell regulation, and the TGFβ signaling pathway, hinting at shared molecular origins. However, each subtype exhibited unique pathways exclusive to them, such as the Hippo signaling pathway for the p53 subtype and cGMP signaling for the *CTNNB1* subtype.

Comparing these findings to the prior literature, studies focusing on endometrial carcinoma yielded different KEGG pathway results. One paper focusing on type I vs. type II endometrial carcinomas highlighted pathways such as p53, lysine degradation, cell cycle, and tight junction, while another paper analyzing endometrial carcinoma vs. normal adjacent mucosal tissue uncovered PI3K-AKT signaling, ECM-receptor interaction, and Focal adhesion [53,54]. KEGG analysis of stage I endometrial carcinomas vs. atrophic endometrium revealed staphylococcus aureus infection, estrogen signaling pathway, IL-17 signaling pathway, Renin secretion, inflammatory response, JAK/STAT3, K-Ras, and TNFα/NF-κB [55]. Collectively, the KEGG results from these studies did not align with our outcome, and this divergence could be attributed to variations in study objectives (molecular subtypes versus normal tissue) and the molecular analysis method employed—(miRNA in this study, mRNA transcripts in previous research).

Notably, a separate study by Widodo et al. exploring differentially regulated miRNAs in endometrial carcinoma demonstrated some overlapping pathways with the current analysis (TGFβ signaling, Glioma, Pathways in cancer), using MirPath [56]. However, their KEGG analysis did not align with the present results, possibly due to differences in analysis method and molecular subtypes investigated.

Among the examined molecular subtypes, the *POLE*-ultra-mutated subtype stood out with distinct KEGG-analyzed signaling pathways, which corresponded to its favorable prognosis compared to the other subtypes [2]. The difference in the identified signaling pathways may provide a plausible explanation for this observation. For example, TGFβ signaling is not highlighted as a prominent signaling pathway in the *POLE*-ultra-mutated subtype. The TGFβ signaling axis supports increased cell growth and proliferation in neoplastic diseases, leading to pronounced aggressiveness and invasion, thus stimulating cancer cell seeding and establishing new metastatic sites [57,58]. Specifically for endometrial carcinomas, TGFβ pathway components undergo deregulation leading to oncogenesis. Impaired expression is observed at every level of signal transduction, beginning from signal induction by TGFβ isoforms, signal reception by plasma membrane receptors and co-receptors, to downstream cytosolic effector Smad proteins [59]. The lack of TGFβ-mediated oncogenic effects in *POLE*-ultra-mutated tumors may explain the less aggressive clinical course for this molecular subtype of EC. At the moment, it is unknown if *POLE* mutation is directly linked to the propensity to avert deregulation of TGFβ signaling in this subtype of endometrial carcinoma. Further studies on this association would be useful.

Another pathway that is prominent in the other three subtypes but not in *POLE*-ultra-mutated tumors is the signaling pathway regulating the pluripotency of stem cells. Cancer stem cells play a pivotal role in cancer progression and drug resistance, and their expression in endometrial carcinomas correlates with tumor aggressiveness and chemoresistance [60]. Pathways known to be involved in cancer stemness are the Hedgehog signaling pathway, PI3K/AKT/mTOR Complex (mTORC) pathway, and NOTCH pathway as well as *Myc* and NF-κB [60,61]. These pathways may be exploited therapeutically for the treatment of the subtypes of endometrial carcinoma that are known to be more aggressive than the *POLE*-ultra-mutated subtype.

Another signaling pathway that is not prominent in the *POLE*-ultra-mutated subtype but present in the other subtypes is fatty acid biosynthesis. The enzyme fatty acid synthase (FASN), responsible for producing long-chain fatty acids, has varying effects on estrogen (E2) signaling in breast and endometrial carcinoma cells [62,63,64]. FASN expression is purported to be part of the estrogen-driven cellular response that leads to proliferation, and inhibiting FASN in endometrial adenocarcinoma cells acts as an antagonist, reducing E2- and tamoxifen-dependent estrogen receptor (ER) transcriptional activity [62]. Furthermore, FASN inhibition led to a marked decrease in E2-stimulated ER expression, suggesting a regulatory role for FASN in controlling ER levels in endometrial carcinoma cells. Notably, this FASN inhibition also contributed to decreased cell proliferation and increased apoptosis, pointing toward its potential as a therapeutic target to modulate endometrial carcinoma progression and hormone-dependent signaling pathways [62]. The involvement of fatty acid biosynthesis as a prominent pathway in the other three molecular subtypes but not the *POLE*-ultra-mutated subtype may thus suggest a biological basis underlying the more aggressive clinical course for these three subtypes.

## 5. Conclusions

In our study, we have demonstrated that different subtypes of endometrial carcinomas, both histological and molecular subtypes, demonstrate differences in miRNA expression. These differences in miRNA expression have implications for diagnosis and prognosis. We also present various signaling pathways involved in the development of endometrial carcinomas—these have possible treatment implications. Tying everything together, miRNAs have the potential to emerge as useful biomarkers for endometrial carcinomas—encompassing diagnosis, treatment, and tailoring treatment.

## Figures and Tables

**Table 1:** Inclusion and exclusion criteria for case selection.

Inclusion Criteria	Exclusion Criteria
Cases from 2008 to 2018 Endometrial carcinoma diagnosed as endometrioid, clear cell, serous, de-differentiated subtypes All cases were reviewed again by the department’s gynecological pathologist to confirm the diagnosis Cases that underwent hysterectomy for confirmation of diagnosis	Cases where the diagnosis was uncertain, either in the original report or upon review by the department’s gynecological pathologist Cases with inadequate material on FFPE Cases where the primary cancer may not be endometrial (e.g., possible ovarian primary with spread to endometrium)

**Table 2:** Characteristics of the 119 cases included in this study.

Tumor Type	(n)
Endometrioid carcinoma	89
Grade 1	33
Grade 2	33
Grade 3	23
Serous carcinoma	8
Clear cell carcinoma	13
De-differentiated carcinoma	9

**Table 3:** Number of tumors obtained for each molecular subtype by following the classification system shown in Figure 2.

Tumor Type	(n)
Endometrioid carcinoma	82
Grade 1	30
Grade 2	29
Grade 3	23
Serous carcinoma	5
Clear cell carcinoma	7
De-differentiated carcinoma	8

**Table 4:** Characteristics of the 102 cases subjected to molecular subtyping.

Molecular Subtype	(n)
MMR-deficient	48
*POLE*-mutated	8
P53 wild type (copy number low)	35
P53 mutated (copy number high)	11

**Table 5:** Characteristics of cases with *CTNNB1* mutations.

Tumor Type	(n)
Endometrioid carcinoma	17 (23.3%)
Grade 1	9 (33.3%)
Grade 2	3 (12.5%)
Grade 3	5 (22.7%)
Serous carcinoma	0 (0%)
Clear cell carcinoma	1 (16.7%)
De-differentiated carcinoma	1 (12.5%)

**Table 6:** Differentially expressed miRNAs between histological subtypes (Only miRNAs with a fold change greater than 0.5 are shown in this table.

Differential Expression of Micro-RNAs between Histological Subtypes (Fold Change)							
Endometrioid		Serous		Clear Cell		De-Differentiated	
**Increased**		**Increased**		**Increased**		**Increased**	
miR-449a miR-449b-5p miR-449c-5p miR-375 miR-34c-5p miR-187-3p miR-190b miR-184 miR-34c-3p miR-17-3p miR-342-5p miR-363-3p miR-568 miR-195-5p miR-147a miR-146a-5p miR-34b-3p miR-163 miR-145-5p miR-526b-5p miR-504-5p	(14.9) (13.5) (7.87) (4.76) (3.42) (3.20) (2.31) (2.13) (2.11) (1.88) (1.81) (1.80) (1.73) (1.72) (1.72) (1.70) (1.70) (1.66) (1.62) (1.52) (1.51)	miR-9-3p miR-125b-5p	(4.26) (1.67)	miR-375 miR-638 miR-1915-3p miR-663a miR-1246 miR-608 miR-612 miR-363-5p miR-765 miR-1290	(4.05) (2.39) (2.43) (1.99) (1.70) (1.69) (1.68) (1.59) (1.58) (1.53)	miR-449a miR-449b-5p miR-449c-5p miR-205-5p miR-10a-5p miR-96-5p miR-182-5p miR-335-5p	(9.68) (8.30) (5.28) (2.99) (2.16) (2.00) (1.83) (1.62)
**Decreased**		**Decreased**		**Decreased**		**Decreased**	
miR-519a-3p miR-301b-3p miR-31-5p miR-590-5p miR-18a-5p miR-18b-5p miR-301a-3p	(0.33) (0.33) (0.34) (0.36) (0.38) (0.49) (0.49)	miR-638	(0.47)	miR-135b-5p miR-30a-3p miR-135a-5p	(0.37) (0.43) (0.44)	miR-638 miR-612 miR-663a	(0.45) (0.45) (0.47)

**Table 7:** Differentially expressed miRNAs between molecular subtypes.

Differential Expression of Micro-RNAs between Molecular Subtypes (Fold Change)							
MMR-D		POLE-Ultra-Mutated		Copy Number High		NSMP (No Specific Molecular Profile)	
**Increased**		**Increased**		**Increased**		**Increased**	
miR-483-3p miR-885-5p miR-1915-3p miR-564 miR-196a-5p miR-16-5p	(1.34) (1.37) (1.37) (1.32) (1.29) (1.24)	miR-139-5p miR-342-3p miR-374b-5p	(1.60) (1.44) (1.36)	miR-34c-5p miR-133b miR-497-5p miR-143-3p miR-145-5p	(2.04) (1.53) (1.43) (1.43) (1.43)	miR-205-5p miR-200a-3p miR-497-5p miR-141-3p miR195-5p	(2.07) (1.51) (1.47) (1.45) (1.35)
**Decreased**		**Decreased**		**Decreased**		**Decreased**	
miR-142-5p miR-301a-3p	(0.73) (0.77)	miR-515-5p miR-210-3p miR-885-3p miR-612 miR-613	(0.31) (0.49) (0.55) (0.55) (0.55)	miR-31-5p miR-31-3p miR-592	(0.30) (0.40) (0.58)	miR-31-5p miR-592 miR-1246	(0.34) (0.52) (0.68)

**Table 8:** miRNAs differentially expressed in *CTNNB1* mutated tumor.

*CTNNB1*
**miRNAs increased**
miR-203a-3p (1.71)
miR-15a-5p (1.29)
miR-16-5p (1.25)
**miRNAs decreased**
miR-499a-5p (0.47)
miR-184 (0.56)
miR-342-5p (0.58)
miR-135a-5p (0.58)

## Data Availability

The data presented in this study are available on request from the corresponding author. The data are not publicly available due to ethical and legal concerns.

## Supplementary Materials

The following supporting information can be downloaded at: https://www.mdpi.com/article/10.3390/cancers15215260/s1, Table S1: List of miRNAs profiled.
